# Editorial: Non-invasive and minimally invasive vagus nerve stimulation for chronic pain

**DOI:** 10.3389/fpain.2024.1402918

**Published:** 2024-05-15

**Authors:** Anna Woodbury, Peter Staats

**Affiliations:** ^1^ Division of Pain Medicine, Department of Anesthesiology, School of Medicine, Emory University, Atlanta, GA, United States; ^2^ Atlanta VA Health Care System, Veterans Health Administration, United States Department of Veterans Affairs, Decatur, GA, United States; ^3^ electroCore LLC, Basking Ridge, NJ, United States

**Keywords:** vagus, non-invasive, pain, functional abdominal pain (FAP), fibromyalgia (FM), gut-brain, opioid use disorder, PENFS

**Editorial on the Research Topic** Non-invasive vagal nerve stimulation for chronic pain

## Introduction

Chronic pain is highly prevalent, and in the United States, it affects >20% of the population with a higher incidence rate than diabetes, depression, and hypertension (1). However, recovery is possible, with about 1 in 10 people becoming pain free in a 1-year period (1). In order to aid in recovery without creating harm related to addiction, dependence, or overdose, better techniques to manage pain and restore function are needed. Noninvasive vagus nerve stimulation has been demonstrated to be effective in treating underlying pain pathology as well as numerous comorbid conditions such as mood and substance use disorders.

Because the vagus nerve is a mixed nerve, it carries both afferent and efferent fibers, controlling visceral organs, but also providing information to the brain from visceral organs and the periphery. Thus, modulation of the vagus nerve at peripheral sites results in central changes. Vagal afferents result in increased parasympathetic activation, improving mood and sleep, and decreasing stress and inflammation through activation of the cholinergic anti-inflammatory pathway (2). Historically, vagus nerve stimulation has been delivered through a surgically-implanted pacemaker-like device and utilized for a range of neurological conditions including refractory epilepsy and depression (3–5). Surgical implantation, however, is associated with a risk of infection, bleeding, and nerve damage and the inherent cost of an implanted device.

More recently, a variety of non-invasive vagus nerve stimulation (nVNS) devices have been developed to modulate the vagus without the added costs and risks of surgery. Two of the primary targets for nVNS in the periphery are at the level of (1) the neck and (2) the ear, and there is evidence that both trans-cervical vagus nerve stimulation (tcVNS) and trans-auricular vagus nerve stimulation (taVNS) can provide benefit in painful conditions (6, 7). Our topic assembles recent discoveries regarding the application of nVNS and percutaneous VNS in difficult-to-treat complex, chronic pain conditions, such as functional abdominal pain in children, headaches, and opioid withdrawal. Within our topic, several studies utilize auricular percutaneous electric nerve field stimulation (PENFS), which may activate several nerves including those in the vagal complex, serving as a percutaneous form of VNS. PENFS is FDA cleared to treat disorders of the gut-brain interaction, which until now had been treated primarily with pharmacotherapy such as anticholinergic drugs (8). Because acetycholine is a primary neurotransmitter for the vagus nerve, which also impacts digestive and other visceral functions, it is plausible that stimulation of the vagus nerve by PENFS can impact functional abdominal pain and other gut-brain disorders, as discussed below.

This editorial synthesizes recent key findings regarding nVNS and PENFS for disorders related to chronic pain.

## Functional abdominal pain in children, adolescents, and young adults

Adolescents are especially susceptible to functional abdominal pain and to the negative effects of pharmacotherapy, including drug overdose. (9–11) Thus, 31 patients 11–18 years old (81% female) utilizing auricular PENFS therapy for disorders of the gut-brain interaction were prospectively studied over a 4 week period (Chogle et al.). Patients in the observational study reported significant reductions in abdominal pain, anxiety, nausea, somatization, and functional disability following PENFS therapy, and parents reported significant improvements in psychosocial and physical function as well as quality of life or their adolescent children (Chogle et al.). A retrospective analysis of 101 patients age 11–21 years old to compare auricular PENFS against standard medical therapy (cyproheptadine or amitriptyline) for functional abdominal pain disorders found that that PENFS (utilized by 48% of patients included) placed weekly over 4 weeks was more effective than cyproheptadine in improving abdominal pain (Santucci et al.). In a prospective, open-label study of thirty children aged 8–18 years old with drug-refractory cyclic vomiting syndrome, auricular PENFS utilized over 6 weeks resulted in statistically significant decreases in abdominal pain index scores (Karrento et al.). It is notable that abdominal pain index scores continued to decrease through follow-up 4–6 months later (Karrento et al.). These studies, together, suggest that PENFS applied to the auricular branch of the vagus nerve is a viable non-pharmacological alternative for the treatment of pain associated with disorders of the gut-brain interaction, and has the potential to create long-term beneficial effects.

## Primary headache disorders

Neuromodulation utilizing nVNS may also be applied to primary headache disorders (not migraine or cluster), which have low prevalence rates and limited options (Villar-Martinez and Goadsby). Individuals suffering from hemicrania continua, paroxysmal hemicrania, short-lasting neuralgiform headache attacks (SUNCT/SUNA), or cough headache who cannot tolerate preventive medication now have an additional nonpharmacologic, low-risk option in nVNS applied to the cervical path of the vagus nerve. The effects of nVNS/tcVNS for primary headache disorders are not fully elucidated, but thought to be mediated in part through a direct alteration in response of the trigeminal and trigeminothalamic neurons via stimulation of vagal afferents, as well as modulation of various neurotransmitters (Villar-Martinez and Goadsby).

## Opioid use disorder

Chronic pain and the opioid epidemic are intimately linked; it is therefore necessary to treat both pain and concomitant opioid use disorder (OUD) to reduce harm and improve quality of life. In a randomized, double-blind study, 20 patients with OUD were assigned to receive either active or sham tcVNS while undergoing opioid withdrawal (Gazi et al.). The active tcVNS group experienced a mean *decrease* in pain following application of the device, while the sham group experienced a mean *increase* in pain scores (Gazi et al.). Thus, nVNS may offer a viable option for decreasing pain in a high risk group of patients with OUD, potentially preventing relapse.

## State of the research and future directions

While this topic covers the utilization of nVNS and PENFS targeting the vagus nerve in several difficult to treat conditions, such as functional abdominal pain, primary headache disorders, and opioid use disorder, there is also potential for its use in fibromyalgia, post-traumatic stress disorder (PTSD), multiple sclerosis, rheumatologic, and inflammatory conditions, myofascial and musculoskeletal pain, and even acute or post-operative pain conditions. Given the anti-inflammatory and neuromodulatory potential of nVNS, current research has only begun to explore its effects. Future studies will further elucidate the mechanism of nVNS for analgesia. More rigorous, randomized, double-blind, sham-controlled studies are necessary to demonstrate efficacy, as well as large-scale pragmatic trials to evaluate the effectiveness of nVNS for specific chronic painful conditions. Given the low risk of this FDA-cleared therapy, it would be reasonable to offer it to patients suffering from chronic pain, especially to those who would like to avoid pharmacotherapy or invasive procedures.
